# Cost-Effective Hyperspectral Transmissometers for Oceanographic Applications: Performance Analysis

**DOI:** 10.3390/s150920967

**Published:** 2015-08-26

**Authors:** Marta Ramírez-Pérez, Rüdiger Röttgers, Elena Torrecilla, Jaume Piera

**Affiliations:** 1 Institute of Marine Sciences (ICM-CSIC), Passeig Marítim de la Barceloneta 37–49, 08003 Barcelona, Spain; E-Mails: torrecilla@icm.csic.es (E.T.); jpiera@icm.csic.es (J.P.); 2 Helmholtz-Zentrum Geesthacht, Max-Planck-Straße 1, 21502 Geesthacht, Germany; E-Mail: rroettgers@hzg.de

**Keywords:** beam transmissometer, hyperspectral, cost-effective, compact, light emitting diodes, uncertainties assessment, thermal management

## Abstract

The recent development of inexpensive, compact hyperspectral transmissometers broadens the research capabilities of oceanographic applications. These developments have been achieved by incorporating technologies such as micro-spectrometers as detectors as well as light emitting diodes (LEDs) as light sources. In this study, we evaluate the performance of the new commercial LED-based hyperspectral transmissometer VIPER (TriOS GmbH, Rastede, Germany), which combines different LEDs to emulate the visible light spectrum, aiming at the determination of attenuation coefficients in coastal environments. For this purpose, experimental uncertainties related to the instrument stability, the effect of ambient light and derived temperature, and salinity correction factors are analyzed. Our results identify some issues related to the thermal management of the LEDs and the contamination of ambient light. Furthermore, the performance of VIPER is validated against other transmissometers through simultaneous field measurements. It is demonstrated that VIPER provides a compact and cost-effective alternative for beam attenuation measurements in coastal waters, but it requires the consideration of several optimizations.

## 1. Introduction

The increasing pressure on aquatic ecosystems by human activities has led to the development of environmental policies and strategies to conserve and manage coastal environments. These strategies demand implementation of water quality monitoring programs at a wide range of temporal and spatial scales [1]. In order to fulfill these demands, it is necessary to develop cost-effective observational techniques that allow us to analyze ecological and environmental parameters at suitable spatial and temporal resolutions. In this context, the use of optical sensors has been demonstrated to offer distinct advantages for oceanographic applications [2,3]. These instruments enable high-frequency measurements and numerous relationships have been already established between optical properties such as attenuation, scattering and turbidity, and several biogeochemical variables in natural waters [2].

In particular, the beam attenuation coefficient is a common and easily measured inherent optical property of natural waters [4]. Transmissometers (beam attenuation meters) are simple to operate and generally do not require sophisticated data correction schemes [5]. These instruments are conceived to measure the intensity loss of a near-parallel light beam along a defined path length in the water sample, as a consequence of light absorption and scattering processes [6]. Transmissometers typically include a light source and a co-aligned photometer at the two ends of the optical path. The design is theoretically simple but it requires the accurate and robust alignment of the light transmitter and detector [7].

Beam attenuation measurements have proven useful for estimating suspended matter concentration [8,9,10], particle size characteristics [11,12,13] and particle composition [14,15] of suspended materials in natural waters. More recently, transmissometers with hyperspectral resolution have been developed which allow us to carry out spectral shape analysis. The use of hyperspectral data to extract valuable environmental and ecological information has been demonstrated for various optical properties, such as remote-sensing reflectance [16,17], particulate absorption [18,19] and underwater radiance spectra [20], as well as for underwater hyperspectral imaging systems [21].

Additionally, the use of modern technologies in the lighting and detection systems has contributed to the development of cost-effective and compact transmissometers. The use of LEDs as light sources offer numerous advantages compared to traditional alternatives such as tungsten bulbs: increased lifetime, lower cost, reduced power consumption, higher brightness, flexible configuration, smaller size, and a wider choice of spectral ranges [22,23]. Furthermore, the LEDs output is stable for different orientations in contrast to the traditional lamps, for which the effective output can slightly vary depending on the position of the filaments relative to the collimation pinhole.

Finally, the use of a set of different LEDs to emulate the full visible light spectrum as well as a miniature photodiode array detector has enabled the combination of these capabilities in the same transmissometer. This property might be important for cost-effective and widespread use in monitoring campaigns. However, the potential advantage of this setup remains to be explored since there are few field studies that employ hyperspectral transmissometers with this novel LED configuration. In the present paper, we examine the operation of a LED-based hyperspectral transmissometer, and compare its performance against two different transmissometers in coastal waters. Based on our measurements, several points of interest for future users and sensor development are discussed.

## 2. Material and Procedures

### 2.1. Instrument Description

**Figure 1:**
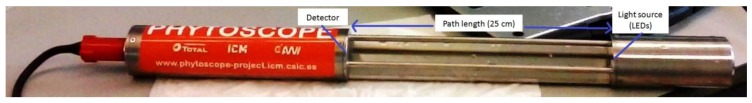
Hyperspectral VIS-Photometer VIPER (TriOS GmbH), path length of 25 cm.

Advances in core photonics and optical instrumentation have led to the development of submersible transmissometers that use LEDs as light source and photodiode arrays as detector with hyperspectral resolution. One such commercially available model is the VIS-Photometer VIPER from TriOS GmbH (Rastede, Germany, Figure 1).

**Figure 2:**
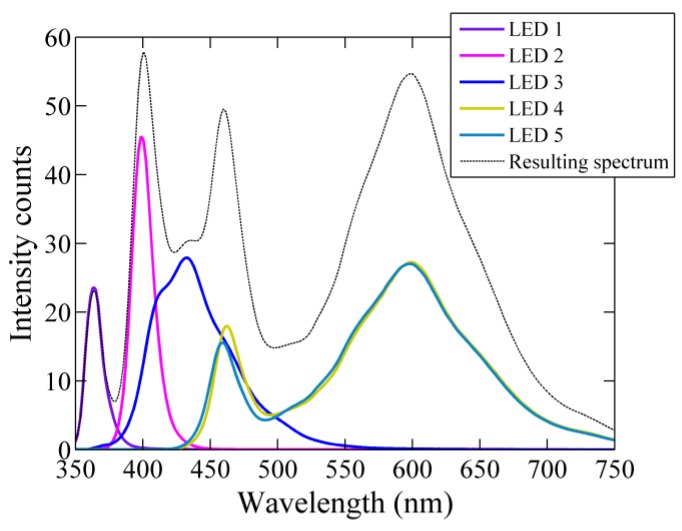
Individual light emission spectra of the 5 LEDs used by VIPER. The emission peak wavelengths, λ_p_, are 365 nm, 400 nm, 430 nm, 600 nm and 600 nm for the LED 1 to LED 5, respectively. The dashed line represents the final emission spectrum (combination of the 5 LEDs).

Due to the limited range of the LEDs emission spectrum, this instrument combines light from five LEDs with different peak wavelengths to emulate a white-light spectral beam (Figure 2). The light beam is collimated with a lens system of three lenses and a pinhole, providing a divergence angle of the beam of 1.33° (pers. comm. TriOS GmbH). The detector is a CMOS array covering the spectral range of 360–750 nm with a spectral resolution of 1.88 nm/pixels and an effective optical resolution of 15 nm (defined by the FWHM). According to the manufacturer, the detector optics provides a light acceptance angle of 0.8° (pers. comm. TriOS GmbH). The path length of the VIPER instrument employed in this study is 25 cm, with a manufacturer rating of suitable attenuation coefficients in the range between 0.04 m^−1^ and 9.2 m^−1^ [24]. VIPER has an open-path design, which avoids the use of a flow tube and an external pump. In addition, VIPER is light-weight and relatively easy to handle for field operations, since the instrument has no internal memory or batteries.

#### Principles of Operation

VIPER measurements are directly influenced by ambient light since this instrument does not use a flow-tube to optically isolate the sample from the surrounding medium [7]. This influence cannot be compensated by using intensity modulation because of the employed type of detector. For this reason, two consecutive measurements of the intensity signal, *V*, are required to correct for ambient light effects. A first signal, *V*_D_, is measured with the light source switched on. A second signal, *V*_D_^dark^, is obtained with the light source switched off. *V*_D_^dark^ measures the light intensity induced by the ambient light alone. The latter signal is subtracted from the first, thereby the dark current of the detector is removed as well:

*V*(λ) = *V*_D_(λ) − *V*_D_^dark^(λ) (1)

*V*(λ) is the final light intensity measurement. The intensity counts are converted into physical units by normalizing by both the maximum number of bits, 2^16^ − 1, and the maximum integration time (IT), 8192 ms. Although the IT can be manually selected, typically it is automatically adjusted by the instrument:

Φ_uncorr_(λ) = [*V*(λ)·8192]/[(2^16^−1)·IT] (2)

Φ_uncorr_(λ) is the spectrum converted to physical units.

A temperature correction for the LED output is then applied to obtain the calibrated spectrum, Φ_T_(λ):

Φ_T_(λ) = Φ*_uncorr_*(λ)/*corr_factor*(λ) (3)

where *corr_factor*(λ) is the correction factor for the LED temperature. Its wavelength dependence was fitted to a quadratic function by the manufacturer:

*corr_factor*(λ) = 1 + c_1_(λ)(*T_LED_* − *T_ref_*) + c_2_(λ)(*T_LED_* − *T_ref_*)^2^ (4)

where c_1_ and c_2_ are the wavelength-dependent coefficients provided by the manufacturer in a calibration file; T_LED_ is the temperature of the LEDs, which is measured by the instrument with an internal temperature sensor, and T_ref_ is the reference temperature recorded during the calibration, 20 °C in this case.

The beam transmittance, *T*(λ), is calculated as the ratio between the flux transmitted to the detector and the flux entering to the water at the source window [7]. In practice, it is difficult to determine the absolute photon flux without a separate reference detector. For this reason, the beam transmission is calculated relative to a reference medium, Φ*_ref_*(λ) (ultrapure water for oceanography):

*T*(λ) = Φ*_T_*(λ)/Φ*_ref_*(λ) (5)

Thereby the attenuation by all water constituents except water itself, *c*(λ), is determined as:

*c(λ) = −LnT(λ)/r* (6)

where *r* is the instrument's path length in meters and *c*(λ), the beam attenuation in m^−1^.

### 2.2. Uncertainty Assessment of the Measurement System

**Figure 3:**
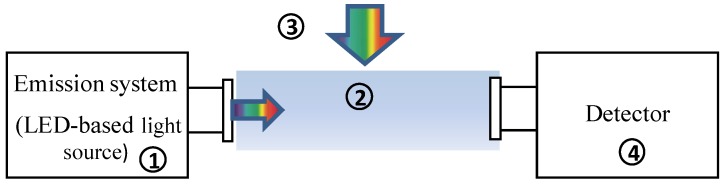
Sources of uncertainties in LED-based hyperspectral open-path transmissometers: (1) Instrument stability; (2) Temperature and salinity dependence of attenuation by water; (3) Effect of ambient light; (4) Temperature sensitivity of the detector and stray light effects.

A proper instrument operation requires a prior knowledge about potential sources of uncertainty. In the case of LED-based hyperspectral open-path transmissometers, uncertainties may derive from different sources (see Figure 3).

(1) A proper instrument operation demands that lighting and detection systems must be stable over time. This stability depends directly on mechanical, electronic, electrical, thermal and optical characteristics of the instrument. The thermal management of the LED light source is one of the most critical factors concerning this issue [25,26]. The performance of a LED is significantly dependent on temperature, which implies a proper heat dissipation and temperature compensation for a good reproducibility of operation. For compensation purposes, the LED temperature is measured by an internal temperature sensor and used to correct the collected spectra by means of correction coefficients provided in a calibration file (see Equation (4)). Other optical sensors, such as the absorption meter a-Sphere (Hobi Labs, Arivaca, AZ, USA), employ a cost-intensive system to regulate the temperature of the LEDs. In this case, the temperature of the light source is set to a specific value by heating/cooling the system [27].

(2) *c*(λ) is calculated relative to a reference medium (*i.e*., pure water), which is assumed to have constant optical properties. However, it is well known that *c*(λ) of pure water depends on temperature and salinity, so these effects have to be corrected by including them in wavelength-dependent correction factors [28,29,30]. Temperature and salinity also induce changes in the real part of the refractive index of both water and the glass material of the sensor’s optical windows (*i.e*., light source and detector windows). For this reason, these correction factors are specific for each instrument.

(3) In open-path transmissometers, the ambient light field is assumed to remain constant within the time period between light and dark measurements. In this way, the effect of the ambient light is subtracted from the transmitted flux. This approach, however, may have problems under high-frequency variability of the ambient light [7] especially when dealing with emission light spectra that are not spectrally flat. In these cases, the sensitivity to ambient light may result stronger and wavelength dependent.

(4) A well-calibrated hyperspectral array-based spectrometer requires a detailed characterization of technical issues such as the temperature sensitivity and the spectral stray light. The VIPER’s detector is a micro-spectrometer from Hamamatsu Photonics K.K. (Hamamatsu, Japan). The technical specifications of this model can be found in the corresponding datasheet [31]. For this reason, these uncertainties have not been analyzed in the present research.

## 3. Assessment

### 3.1. Instrument Stability

#### 3.1.1. Instrument Precision

**Figure 4:**
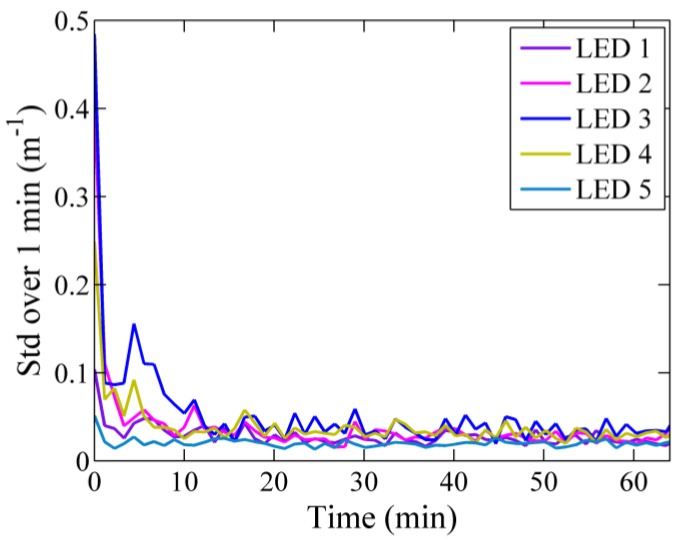
Stability of the VIPER output signal over one hour. Given is the standard deviation of the attenuation coefficient for 15 consecutive measurements (one min) collected in air.

The stability of the VIPER output signal was analyzed in the laboratory. With this purpose, measurements of light intensity were collected in air for one hour. Similarly to Sabbah *et al.* [32], the precision of the measurement was determined by calculating the standard deviation within one-minute intervals (approximately 15 readings). The results obtained at the peak wavelengths of the different LEDs are shown in Figure 4. Typically, all light sources require a warm-up period to reach a stable output, which was about 10 min in the case of VIPER. After this period, the signal became more stable at all examined wavelengths, leading to a minimum standard deviation of *c*(λ) of 0.02 m^−1^ at 700 nm and a maximum value of 0.04 m^−1^ at 430 nm.

#### 3.1.2. Thermal Management

**Figure 5:**
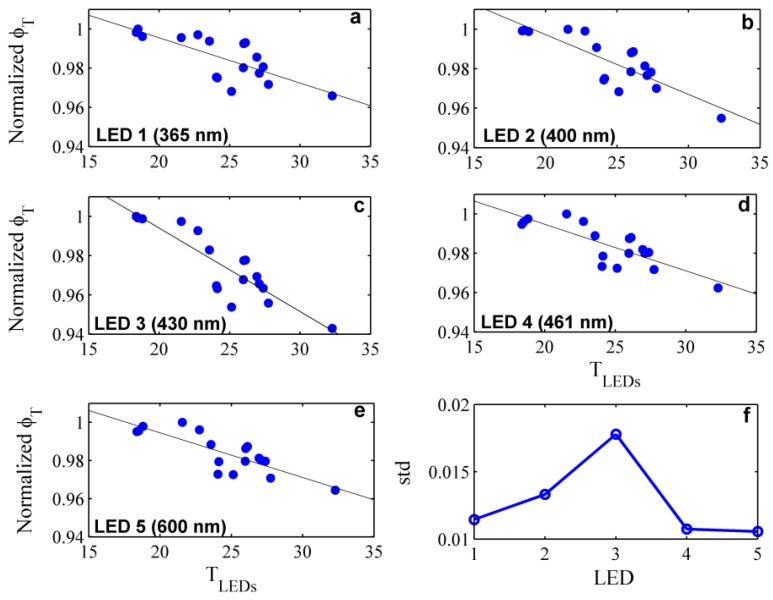
(**a**–**e**) Normalized transmitted light intensity as function of the operating temperature at the emission peak wavelengths of the five LEDs (*i.e*., 365 nm, 400 nm, 430 nm, 461 nm and 600 nm, respectively). The linear regression slope indicates the rate of change in the corrected output signal with the temperature of the diode; (**f**) Standard deviation of the measured data for the five LEDs.

The dependency of the fully corrected output signal on the temperature of the LEDs was examined within the temperature range from 18 °C to 35 °C. With this aim, the instrument was immersed in purified water at different water temperatures and after the warm-up period, the transmitted light was recorded continuously for a couple of minutes. Data were corrected for both, LED-temperature effects and water temperature dependencies (see details in Section 3.2.1). The variability in the corrected transmitted light with the operating temperature of the LEDs was fitted with a simple linear regression model (Figure 5a–e). In addition, the standard deviation was calculated in order to quantify this effect (Figure 5f). The results showed that, regardless of the LED-temperature correction (Equations (3) and (4)), the light intensity decreased with increasing operating temperature. This is the expected behavior when the temperature dependence is not accurately corrected [33]. In particular, these variations were larger at LED 3 with the emission peak at 430 nm, where both the rate of change in light intensity and the standard deviation were significantly higher (Figure 5c,f).

**Figure 6:**
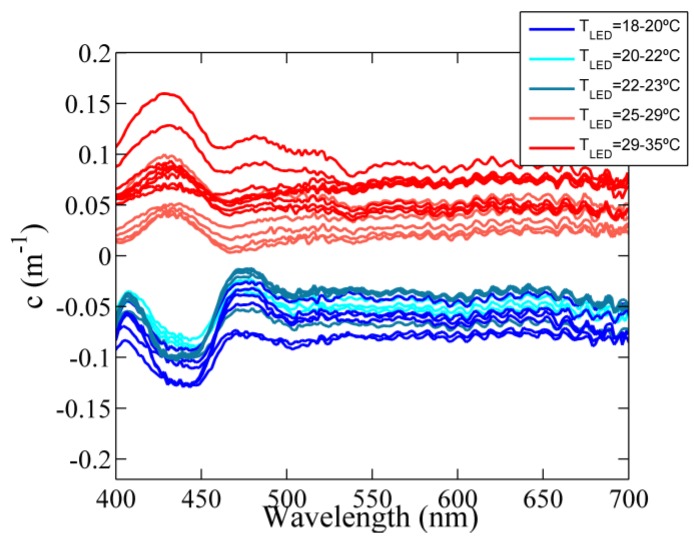
Corrected beam attenuation spectrum of purified water at different LED temperatures. The attenuation spectra were calculated relative to a reference spectrum with a LED-temperature of 24 °C.

To assess the effects of the observed fluctuations on the final beam attenuation coefficient, the measurements were processed using the same reference spectrum with a LED-temperature of 24 °C (Figure 6). The results showed that not only the magnitude but also the spectral shape of the beam attenuation coefficient varied as a function of the LED-temperature. A change of 0.17 m^−1^ was obtained for a LED-temperature increment of 17 °C at 600 nm, which implied an error of 0.01 m^−1^/°C. In addition, anomalous spectral features were observed from 420 nm to 440 nm (LED 3, see Figure 2), characterized by negative values of the beam attenuation when the LED-temperature of the sample was cooler than the reference and *vice versa* (blue and red lines in Figure 6, respectively).

### 3.2. Instrument-Specific Temperature and Salinity Correction Factors

Laboratory experiments were carried out to determine the VIPER-specific temperature and salinity correction factors for the pure water attenuation coefficient. They are used in a correction procedure similar to that one employed by Pegau *et al.* [28], Sullivan *et al.* [30] and Röttgers *et al.* [34], for the AC-9, AC-S and PSICAM instruments, respectively.

The temperature and salinity dependence analysis was performed by using purified water (Milli-Q) at temperatures between 10 °C and 35 °C and a concentrated NaCl solution of 200 g/kg, respectively. The corresponding correction factors, ψ^i^_T_ and ψ^i^_S_, represent the absolute change in water attenuation coefficient with the variation in temperature and salinity, *i.e*., δ*c*(λ)/δT and δ*c*(λ)/δS (units (m^−1^·°C^−1^) and (m^−1^·S‰^−1^), respectively).

In addition, the results were compared to model simulations of optical density in a cuvette based on Max and Chapados [35]. Additional details about the experiments, model simulations and the obtained temperature and salinity correction factors are provided in Appendix A.

#### 3.2.1. Temperature Correction Coefficients

**Figure 7:**
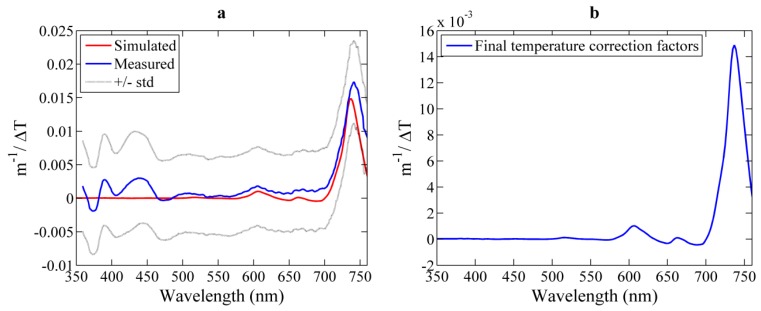
(**a**) Results from the temperature dependence analysis and the comparison with model simulations based on the approach by Max and Chapados [35] (red line). The blue line corresponds to the average over the 20 experimental runs and the standard deviation is represented with dotted line; (**b**) Final VIPER-specific temperature correction coefficient for pure water beam attenuation as a function of the wavelength.

VIPER-derived ψ^i^_T_ spectral values and those predicted by model simulations are given in Figure 7a. The spectral features for λ > 500 nm agree very well with previous studies [28,29,30] and with the model (*i.e*., two smaller peaks at 604 and 662 nm and a large one at 740 nm), mainly showing the influence of temperature on the pure water light absorption. Spectral features induced by temperature changes at shorter wavelengths (λ < 500 nm) are very small [28,29,30], and changes in the scattering coefficient here should not display any spectral signature, except a typical exponential increase with deceasing wavelength [29]. Hence, the spectral fluctuations observed in VIPER results at <500 nm were considered to be induced by the above mentioned poor temperature compensation at the LED 3 (see Section 3.1.2). The small offset between VIPER measurements and model predictions was in the range of the experimental errors. When ignoring the spectral features at shorter wavelengths, the experimentally obtained ψ^i^_T_ can be well represented by the model results of the specific optical setup. Thus, ψ^i^_T_ values predicted by the model are considered here as final temperature correction factors (Figure 7b and Table A1 in the Appendix A).

#### 3.2.2. Salinity Correction Coefficients

**Figure 8:**
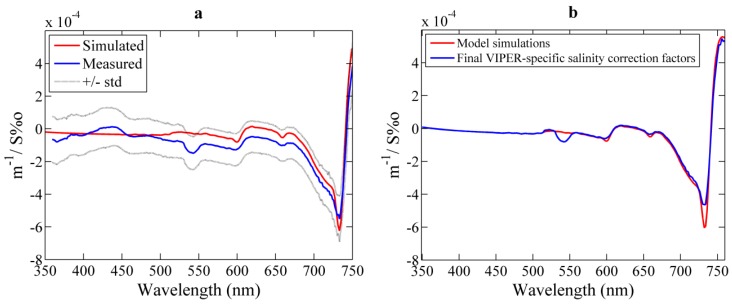
(**a**) Results from the salinity dependence analysis and comparison with model simulations based on the Max and Chapados [35] approach (red line). The blue line corresponds to the average over 20 experimental runs and its standard deviation is represented by dotted lines; (**b**) Final VIPER-specific ψ^i^_S_ spectrum for pure water beam attenuation as a function of the wavelength (blue line) and comparison with model simulations (red line).

The effects of the concentration of ions on the water attenuation and the optical path transmission were obtained both experimentally and computationally by using NaCl to represent seawater (Figure 8a). The ψ^i^_S_ spectral values derived from both methods concurred for the spectral region λ > 550 nm and show overall agreement with previous works [29,30]. The main spectral features were the small negative troughs at 598 and 660 nm and the deeper negative trough at 733 nm followed by the positive peak at 757 nm. However, the noticeable trough at 540 nm displayed by VIPER results was not observed by model simulations or other studies. This behavior might be caused by stray light artifacts, whose analysis is outside the scope of the present work. Nevertheless, this effect is considered as an instrument-specific spectral feature since it was observed in all the experimental runs. The results for 360–500 nm showed an anomalous behavior that varied along the different tests. Similar to the results of the temperature analysis, it was assumed that this behavior is related to the poor temperature compensation of the LED 3. Thus, these spectral features were discarded in the final ψ^i^_S_ spectrum, and instead the model results were used here. Wavelength-independent differences between measured and modeled ψ^i^_S_ were again smaller than the experimental error. For wavelengths >500 nm the obtained ψ^i^_S_ spectrum was finally adjusted to the level of the model results as depicted in Figure 8b. Small differences on the magnitude of the troughs are due to the difference in optical resolution, which is 15 nm for VIPER and 1 nm for the model.

### 3.3. Ambient Light Effects

The instrument’s sensitivity to ambient light fluctuations was analyzed in the field under different deployment conditions and different distances from the water surface. The study was performed on two different areas: Lake Schaalsee in northern Germany and Alfacs Bay in the NW Mediterranean coast (Spain). Sampling in Schaalsee was characterized by calm wave conditions and performed with the instrument hanging from a floating buoy. On the contrary, measurements in Alfacs Bay were conducted under rough sea surface conditions and the instrument was deployed from a winch mounted on a small boat.

**Figure 9:**
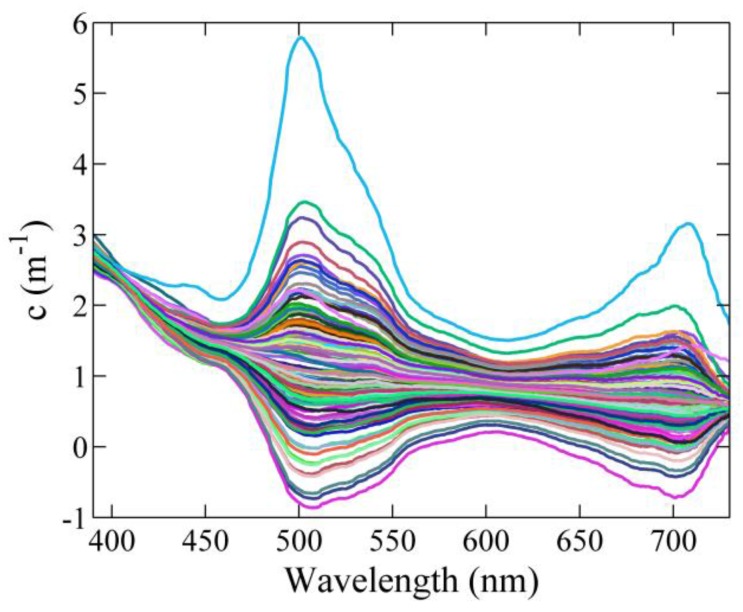
Example of artifacts in the measurements at specific spectral regions due to ambient light contamination during floating buoy deployment in Schaalsee Lake (Germany) in September 2014.

**Figure 10:**
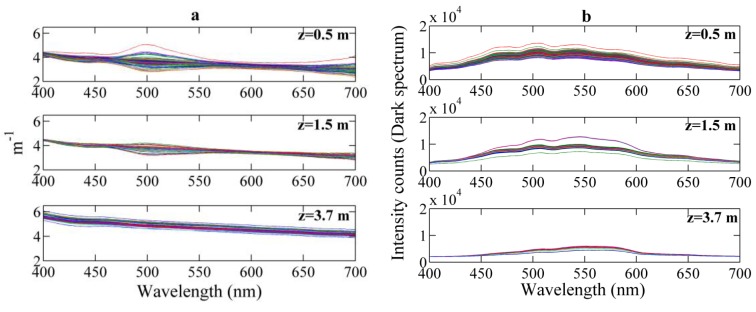
(**a**) Beam attenuation and (**b**) dark spectrum measured for several minutes at different depths in Alfacs Bay (NW Mediterranean coast, Spain) in March 2014.

The beam attenuation spectra measured under both deployment conditions showed spectral contamination due to high-frequency variations in the ambient light (see Figure 9 and Figure 10). In both cases, the intensity collected by the instrument along the water column was slightly modified by waves focusing effects of sunlight occurring at periods of time shorter than the time interval between “dark” and light measurements. The induced artifacts were observed at specific wavelengths (500–550 nm and ~700 nm), at which the light intensity emitted by the LED system has minima (Figure 2). This causes that variations in ambient light conditions have a relatively large significant effect.

The observed contamination also depends on the ambient light intensity, and consequently, on the sampling depth and turbidity level. Figure 10 shows the beam attenuation and the corresponding environmental light conditions (spectra measured with the light off) collected at different depths in a fixed station in Alfacs Bay. The variations in ambient light had a significant influence close to the surface, but decreased with water depth, being negligible at 3.7 m below the water surface.

### 3.4. Instrument Performance during in Situ Measurements

The suitability of the VIPER transmissometer for coastal oceanographic applications was evaluated by comparison with other *in situ* transmissometers widely used and validated by the scientific community (AC-S, from WET Labs and LISST 100-X, from Sequoia Scientific Inc., Bellevue, WA, USA). AC-S is a double cuvette spectrophotometer to measure the absorption and attenuation coefficients. It is a hyperspectral sensor (400 to 750 nm) with a spectral resolution of 5 nm, a band pass of 14–18 nm and the attenuation channel has a receptor acceptance angle of 0.93°. It employs flow tubes, tungsten lamps as light source and broadband detectors. Light of different wavelengths is analyzed by passing the collimated beam through a rotating, linear variable interference filter onto the detector [36]. The LISST 100-X is designed to measure volume scattering in near-forward direction at 32 different angles to determine the particle size distribution. It also measures the beam transmission at 670 nm along a 5-cm path length using a highly collimated, monochromatic and polarized light source, and the detector’s acceptance angle is 0.0269° [37].

The comparison was performed with the beam attenuation coefficients measured by (1) 25-cm path length VIPER; (2) 25-cm path length AC-S and (3) 5-cm path length LISST 100-X. For this analysis, data collected during two field campaigns at a fixed station in the shallow estuarine area of Alfacs Bay (NW Mediterranean coast, Spain) were used. The dataset consisted of 24 h time series of vertical profiles from 0.5 m to 3.5 m (maximum depth) with 0.5 m depth intervals. At each depth level, optical sensors were sampling continuously for five min.

#### 3.4.1. VIPER *vs.* AC-S

**Figure 11:**
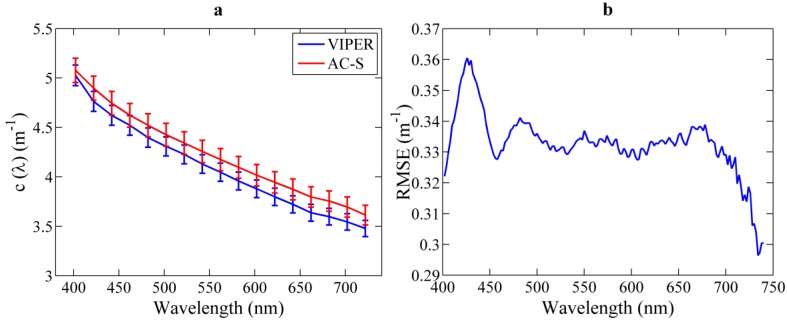
(**a**) Mean average and standard error of the spectral beam attenuation measured by VIPER (blue) and by AC-S (red) in Alfacs Bay, respectively; (**b**) Root mean square error between VIPER and AC-S at different wavelengths for the analyzed data.

For the comparison of VIPER and AC-S, simultaneous measurements were collected from 6 p.m. on 24 March to 6 p.m. on 25 March 2014 in Alfacs Bay. For both instruments, recorded data were corrected for temperature and salinity effects and were averaged over five minutes. Due to the possibility that the presence of air bubbles in the path deteriorates the signal, only spectra with a relative error lower than 0.1 at 400 nm were selected for the analysis, which corresponded to about 60% of the total number of measurements. In general, good overall agreement was found between both instruments, although at 600 nm the attenuation coefficient was, on average, 4% higher for AC-S than for VIPER (see Figure 11a). To evaluate the statistical significance of the difference in the beam attenuation of both instruments, a Student’s two-tailed *t-*test was applied. Based on these results, no significant differences were found over the complete wavelength range investigated (*p* = 0.77 on average). Nevertheless, the root mean square error (RMSE) between both instruments was calculated as a function of the wavelength (see Figure 11b). The maximum RMSE, 0.36 m^−1^, was obtained at 430 nm strongly suggesting that artifacts associated with the thermal management of LED-430 nm are responsible for this behavior.

**Figure 12:**
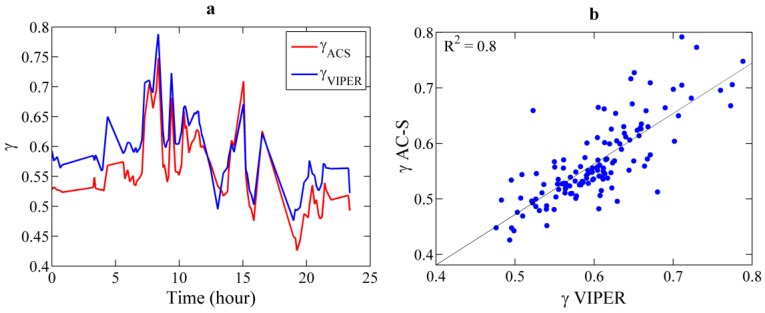
(**a**) Comparison of beam attenuation slopes, γ, measured with VIPER (blue) and AC-S (red) during vertical profiling at a fixed station in Alfacs Bay in March 2014; (**b**) Linear correlation between the slopes derived from both instruments for the analyzed time series.

For a more detailed assessment of the different spectral shapes derived from both instruments, the spectral slopes were compared. For this analysis, beam attenuation spectra were fitted to a power law function *c*(λ) = A·λ^−γ^ between 400 and 700 nm. The goodness of fit, *R^2^*, was higher than 95% in all cases and the RMSE was of 0.036 and 0.034 for VIPER and AC-S, respectively. VIPER and AC-S spectral slopes exhibited a good correlation (*R^2^* = 0.8) and the same temporal trend along the time series, although VIPER slopes were slightly steeper than those from the AC-S (Figure 12). The parameter γ varied between 0.43 and 0.75 for AC-S, whereas for VIPER it varied from 0.48 to 0.79, *i.e*., values about 10% higher in the latter case. However, the result from the *t*-test showed that these differences are not statistically significant, with a p-value equal to 0.12.

#### 3.4.2. VIPER *vs.* LISST 100X

**Figure 13:**
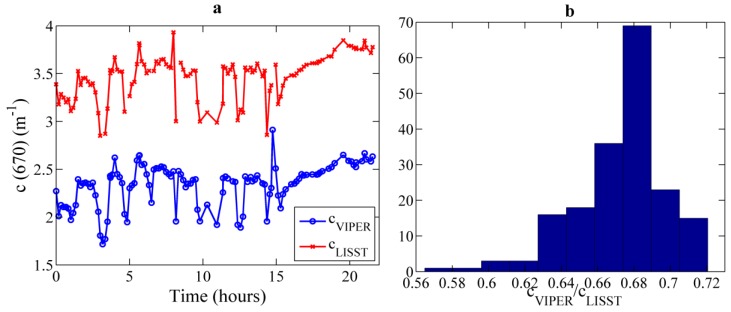
(**a**) Comparison of beam attenuation at 670 nm measured by VIPER (blue) and by LISST-100X (red) during vertical profiling at a fixed station in Alfacs Bay in June 2013; (**b**) Histogram with the frequency of occurrence of the c_VIPER_/c_LISST_ ratio.

A second dataset of experimental measurements was obtained to compare the performance of VIPER with LISST-100X. In this case, simultaneous measurements were collected from 9 p.m. on 24 June to 7 p.m. on 25 June 2013 in Alfacs Bay, and were averaged over five minutes. The beam attenuation at 670 nm exhibited the same temporal trend for both instruments, although its magnitude differed significantly due to the strong difference in the acceptance angle (0.8° *vs.* 0.0269°) [37] (see Figure 13a). The ratio of VIPER to LISST beam attenuation was on average 0.67, *i.e*., VIPER underestimated the beam attenuation by 33% compared to LISST, due to the higher collection of near forward scattered light by VIPER (Figure 13b). This ratio varied between 0.57 and 0.72 along the time series, probably as a consequence of variations in the particle size distribution [37] that changes the volume scattering function. Despite the known effect of the acceptance angle in the absolute beam attenuation coefficient, temporal variations are represented in the VIPER signal as good as in the LISST one.

## 4. Discussion

The performance analysis of the newly-developed VIPER transmissometer allowed us to define the instrument limitations as well as to determine the suitability of this instrument for specific coastal applications.

Firstly, our results suggested that the application range of the VIPER 25-cm path length should be narrower than that one proposed by the manufacturer (*i.e*., from 0.04 m^−1^ to 9.2 m^−1^). Regarding the lower bound, fluctuations of the light intensity were of the order of 0.04 m^−1^ at the shortest wavelengths, which implies an error of about 100% at these attenuation levels. These results were obtained after the warm-up period, which was found here to be around 10 min. Furthermore, according to Bugnolo [38], about 67% of the light will be multiply scattered along a path length of 25 cm at c = 9.2 m^−1^. Therefore, the suitable upper bound should be lower.

Secondly, contrary to the proposal by Voss and Austin [39], the acceptance angle of the VIPER's detector is smaller than the divergence of the beam source. This implies that part of the light exiting the source is not collected by the receiver. However, this light could be partially scattered by the suspended particles and redirected onto the detector. Thus, the real attenuation is underestimated. This explains the differences on the magnitude obtained between VIPER and both, the AC-S and LISST-100X instruments. In the first case, the AC-S beam attenuation coefficient was on average 4% higher than that obtained with VIPER. In the second case, the average VIPER to LISST ratio amounted to 0.67, which was in good agreement to the C-Star to LISST-100X ratio, C_C-STAR_/C_LISST_ = 0.7, found by Boss *et al*. [37] (the C-Star acceptance angle is 1.2°).

Despite these differences on the magnitude of the beam attenuation coefficient, we have demonstrated that VIPER provides reliable measurements in coastal waters based on our comparison to results obtained with the other transmissometers. Our validation was performed under field conditions with an attenuation range between 1.6 m^−1^ and 6 m^−1^. Although no statistically significant differences were found at any of the analyzed wavelengths, the maximum error between VIPER and AC-S was obtained for the spectral range 420–440 nm. An anomalous behavior at this spectral region was also observed in the laboratory tests performed to determine the instrument-specific temperature and salinity correction factors. We observed that the LED-temperature compensation was poorly corrected at these wavelengths. The LED emitting light in this range has an emission peak at 430 nm (LED 3, see Figure 2). In particular, this is a Surface Mounted Design (SMD) LED with ball lens, located at the center of the LEDs array (pers. comm. TriOS GmbH). The anomalous results in this spectral range suggest that this LED is overheated due to its mounting configuration. Depending on their design and position, the LEDs are heated up in a different rate, which is difficult to be accurately measured with a single internal temperature sensor. It is important to mention that these results were obtained in the lab by sampling in a continuous mode. Therefore the use of a longer timer mode might improve the LED-temperature compensation. In this way, the LED bulbs would heat up at a lower rate having a longer time for temperature stabilization.

In addition, some limiting issues related to ambient light contamination were detected at specific deployment conditions. They are a consequence of the open-path design of VIPER and the non-flat white light spectrum emitted by the LEDs. Thus, specific spectral regions present higher sensitivity to slight variations in the environmental conditions, which induce significant spectral artifacts.

However, the constant evolution in LEDs technology led to the development of flat-white LEDs [40]. Therefore, the observed limitations could be significantly avoided in future instrument generations. The LED-temperature compensation could be also improved by the integration of several internal temperature sensors to make possible an accurate measurement of the temperature of each LED. In case that these corrections would not be sufficient, an active temperature regulation system could be implemented to set the LED at a reference temperature. Regarding the ambient light contamination, these effects could be somewhat reduced by shielding the instrument from the ambient light.

By considering the recommendations and application limits analyzed in this study, we conclude that the VIPER transmissometer is a potentially interesting tool for specific coastal applications due to the numerous advantages of his design. Furthermore, the distinct features of this instrument allow it to be mounted on several oceanic observing platforms (e.g., gliders, AUVs, mooring systems) for large-scale characterization of oceanic processes.

## 5. Conclusions

A performance analysis was carried out in order to examine the reliability of the beam attenuation coefficient derived from a newly-developed cost-effective hyperspectral transmissometer (*i.e*., the VIS-Photometer VIPER, TriOS GmbH). A distinct feature of this instrument is to employ an array of different LEDs as light source to provide a full visible-light spectrum. In addition, it uses a micro-spectrometer as detector, which makes it compact. The comparison with other commercial transmissometers demonstrated that VIPER provides reliable beam attenuation measurements in coastal waters applications. In addition to these overall satisfactory results, some spectral artifacts were detected at wavelengths in the range 420–440 nm. The origin of this behavior was related to a poor temperature compensation of one of the integrated LEDs (λ_p_ = 430 nm). Ambient light contamination was also observed as a consequence of the non-uniformity in the emitted light intensity, which causes that some spectral regions are more sensitive to the environmental light. These results suggested that future instrument developments should focus on the optimization of these issues. Additionally, a second generation of this instrument should benefit from including a detector acceptance angle equal or higher than the divergence of the beam source. However, with a prior knowledge of the described limiting factors and recommendations of use, the VIPER instrument, as a cost-effective and compact hyperspectral transmissometer, can be considered as a powerful alternative to traditional transmissometers for specific coastal waters applications.

## Appendix

The laboratory experiments conducted to determine the VIPER-specific temperature and salinity dependencies and model simulations were presented in Section 3.2. Nevertheless, an extended description and the tabulated values of the temperature and salinity coefficients (Table A1) are provided in this appendix. All laboratory tests were carried out inside a cleaning blench, which provides particle-free air to protect the purified water from contamination during the experiments. The instrument was connected one hour before starting the experiments in order to have enough time for stabilization.

## A. Model Simulations

The computational model of optical density in a cuvette based on Max and Chapados [35] uses the complex refractive index of the glass cuvette and the medium (water) with its variations with temperature and salinity. The refractive index, and its temperature dependence, of the window glass are taken from the manufacturer’s description (Heraeus quartz glass Suprasil, Hanau, Germany). The water absorption is taken from Pope and Fry [41], the temperature and salinity dependence of water absorption from Röttgers *et al.* [29], and the water scattering, its temperature-dependence, and the influence of the NaCl concentration is calculated according to Zhang *et al.* [42].

## B. Temperature Correction Coefficients

Ten 1-L glass bottles were filled up with pure water. Five of them were heated in a water bath until reaching ~35 °C and the remaining five were cooled in the refrigerator until a temperature of ~10 °C. Then, five tests with four repetitions (*i.e*., 20 runs in total) were performed, which consisted of measuring the attenuation difference spectrum (10 °C *vs*. 35 °C) of two of these samples for three minutes. The first minute of measurements was used just as warm-up period, and the average over the remaining two minutes was performed and the result divided by the difference in temperature between both samples. The changes in water temperature were continually recorded and were also averaged over the two minutes. The final ψ*i*_T_ spectral values were obtained by averaging the 20 experimental runs and were compared to model simulations.

## C. Salinity Correction Coefficients

The NaCl solution was prepared by diluting 100 grs (tests 1 and 2) and 200 gr (tests 3 and 4) of the *EMSURE*^®^ NaCl product (Millipore, Billerica, MA, USA) in 1 L of purified water. Then, these dilutions were vacuum filtered using a three times pre-rinsed 0.22 μm GSWP Millipore filters in order to remove particles. A second filtration was performed at lower pressures (~60 mbar) with the aim of removing possible air bubbles [29]. The experiment started once the sample temperature was stabilized into the cleaning bench and there were no significant differences with respect to the temperature of the reference. The experiment consisted of four tests, with five repetitions per test. Prior to starting each experimental run, reference spectra were also measured in order to control the contamination. Then, beam attenuation measurements of the NaCl solution were collected continuously for three minutes. The first minute was used as warm-up period and the mean average over the two remaining minutes was calculated for a subsequent analysis.

**Table A1:** Hyperspectral temperature (ψ^*i*^_T_, m^−1^ °C^−1^) and salinity (ψ^*i*^_S_, m^−1^∙S^−1^) dependencies for the attenuation of pure water measured with VIPER.

λ	ψ_T_	σ_T_	ψ_S_	σ_S_	λ	ψ_T_	σ_T_	ψ_S_	σ_S_	λ	ψ_T_	σ_T_	ψ_S_	σ_S_
(nm)	(m^−1^·°C^−1^)	(m^−1^·°C^−1^)	(m^−1^·S^−1^)	(m^−1^·S^−1^)	(nm)	(m^−1^·°C^−1^)	(m^−1^·°C^−1^)	(m^−1^·S^−1^)	(m^−1^·S^−1^)	(nm)	(m^−1^·°C^−1^)	(m^−1^·°C^−1^)	(m^−1^·S^−1^)	(m^−1^·S^−1^)
350	3.65E−05	7.97E−03	−1.96E−05	1.67E−04	488	6.90E−07	7.02E−03	−3.71E−05	1.02E−04	626	2.72E−04	6.98E−03	1.61E−05	9.59E−05
352	3.62E−05	8.04E−03	−2.01E−05	1.82E−04	490	1.41E−06	6.99E−03	−3.64E−05	1.01E−04	628	1.83E−04	7.00E−03	1.48E−05	9.66E−05
354	3.60E−05	7.66E−03	−2.07E−05	1.51E−04	492	3.02E−06	6.98E−03	−3.72E−05	1.01E−04	630	1.08E−04	6.99E−03	1.33E−05	9.51E−05
356	3.61E−05	7.68E−03	−2.12E−05	1.51E−04	494	6.25E−06	6.92E−03	−3.77E−05	1.03E−04	632	4.34E−05	6.92E−03	1.08E−05	9.74E−05
358	3.60E−05	7.73E−03	−2.17E−05	1.43E−04	496	1.14E−05	6.93E−03	−3.77E−05	1.04E−04	634	−1.56E−05	6.86E−03	9.81E−06	9.54E−05
360	3.59E−05	7.83E−03	−2.22E−05	1.41E−04	498	1.67E−05	7.03E−03	−3.83E−05	1.02E−04	636	−7.42E−05	6.83E−03	8.75E−06	9.47E−05
362	3.58E−05	7.78E−03	−2.27E−05	1.41E−04	500	2.24E−05	6.95E−03	−3.82E−05	1.02E−04	638	−1.30E−04	6.86E−03	7.52E−06	9.69E−05
364	3.58E−05	7.69E−03	−2.32E−05	1.39E−04	502	2.91E−05	6.88E−03	−3.78E−05	1.02E−04	640	−1.79E−04	6.87E−03	6.24E−06	9.58E−05
366	3.57E−05	7.60E−03	−2.36E−05	1.36E−04	504	3.77E−05	6.85E−03	−3.57E−05	1.04E−04	642	−2.16E−04	6.87E−03	1.20E−06	9.39E−05
368	3.55E−05	7.57E−03	−2.41E−05	1.33E−04	506	4.96E−05	6.94E−03	−3.40E−05	1.00E−04	644	−2.46E−04	6.85E−03	−2.18E−06	9.62E−05
370	3.55E−05	7.62E−03	−2.45E−05	1.31E−04	508	6.65E−05	6.94E−03	−3.43E−05	1.03E−04	646	−2.79E−04	6.85E−03	−5.50E−06	9.78E−05
372	3.54E−05	7.62E−03	−2.49E−05	1.29E−04	510	8.75E−05	6.92E−03	−3.46E−05	1.06E−04	648	−3.07E−04	6.83E−03	−8.78E−06	9.50E−05
374	3.54E−05	7.70E−03	−2.53E−05	1.32E−04	512	1.08E−04	6.90E−03	−3.36E−05	1.04E−04	650	−3.16E−04	6.89E−03	−1.07E−05	9.54E−05
376	3.64E−05	7.59E−03	−2.57E−05	1.26E−04	514	1.22E−04	6.89E−03	−2.83E−05	9.80E−05	652	−2.95E−04	6.93E−03	−1.74E−05	9.50E−05
378	4.32E−05	7.62E−03	−2.61E−05	1.22E−04	516	1.28E−04	6.87E−03	−2.48E−05	1.01E−04	654	−2.38E−04	6.92E−03	−2.61E−05	9.46E−05
380	4.91E−05	7.79E−03	−2.65E−05	1.26E−04	518	1.24E−04	6.92E−03	−2.24E−05	9.97E−05	656	−1.46E−04	6.90E−03	−3.21E−05	9.65E−05
382	4.72E−05	7.68E−03	−2.68E−05	1.20E−04	520	1.15E−04	6.89E−03	−2.19E−05	9.95E−05	658	−3.56E−05	6.91E−03	−3.61E−05	9.51E−05
384	3.69E−05	7.80E−03	−2.72E−05	1.25E−04	522	1.02E−04	6.87E−03	−2.10E−05	1.03E−04	660	5.61E−05	6.97E−03	−3.27E−05	9.58E−05
386	3.47E−05	7.82E−03	−2.75E−05	1.22E−04	524	8.97E−05	6.89E−03	−1.98E−05	1.03E−04	662	1.03E−04	7.04E−03	−3.23E−05	9.77E−05
388	4.04E−05	7.88E−03	−2.78E−05	1.20E−04	526	7.70E−05	6.85E−03	−1.99E−05	1.00E−04	664	1.01E−04	6.99E−03	−2.40E−05	9.87E−05
390	4.48E−05	7.88E−03	−2.82E−05	1.23E−04	528	6.41E−05	6.86E−03	−1.31E−05	1.01E−04	666	6.84E−05	6.95E−03	−1.91E−05	9.83E−05
392	4.04E−05	7.83E−03	−2.85E−05	1.24E−04	530	5.13E−05	6.90E−03	−1.93E−05	9.72E−05	668	2.13E−05	6.97E−03	−2.19E−05	9.56E−05
394	3.35E−05	7.72E−03	−2.88E−05	1.23E−04	532	3.96E−05	6.84E−03	−4.23E−05	1.01E−04	670	−2.98E−05	7.06E−03	−2.06E−05	9.60E−05
396	2.89E−05	7.62E−03	−2.90E−05	1.21E−04	534	3.01E−05	6.82E−03	−5.71E−05	9.96E−05	672	−9.00E−05	7.18E−03	−2.08E−05	9.58E−05
398	2.68E−05	7.51E−03	−2.93E−05	1.19E−04	536	2.30E−05	6.73E−03	−6.82E−05	1.02E−04	674	−1.62E−04	7.16E−03	−2.59E−05	1.00E−04
400	2.75E−05	7.41E−03	−2.96E−05	1.19E−04	538	1.75E−05	6.70E−03	−7.55E−05	1.01E−04	676	−2.38E−04	7.03E−03	−3.18E−05	9.64E−05
402	2.88E−05	7.37E−03	−2.98E−05	1.17E−04	540	1.31E−05	6.74E−03	−7.83E−05	1.02E−04	678	−3.01E−04	7.06E−03	−4.07E−05	9.74E−05
404	2.65E−05	7.38E−03	−3.01E−05	1.16E−04	542	8.29E−06	6.77E−03	−8.14E−05	1.01E−04	680	−3.44E−04	7.07E−03	−5.15E−05	9.60E−05
406	1.85E−05	7.40E−03	−3.03E−05	1.14E−04	544	3.65E−06	6.76E−03	−7.95E−05	1.01E−04	682	−3.75E−04	7.13E−03	−5.85E−05	9.86E−05
408	1.11E−05	7.43E−03	−3.06E−05	1.16E−04	546	2.16E−07	6.87E−03	−7.22E−05	9.94E−05	684	−4.02E−04	7.12E−03	−6.83E−05	9.57E−05
410	1.00E−05	7.51E−03	−3.08E−05	1.14E−04	548	−4.10E−07	6.93E−03	−6.26E−05	9.75E−05	686	−4.23E−04	7.20E−03	−8.13E−05	9.66E−05
412	1.66E−05	7.57E−03	−3.10E−05	1.15E−04	550	5.78E−07	6.93E−03	−5.18E−05	9.98E−05	688	−4.31E−04	7.14E−03	−9.46E−05	9.84E−05
414	2.39E−05	7.68E−03	−3.13E−05	1.16E−04	552	6.73E−08	6.97E−03	−3.79E−05	9.75E−05	690	−4.25E−04	7.05E−03	−1.08E−04	1.01E−04
416	2.45E−05	7.74E−03	−3.15E−05	1.17E−04	554	−3.81E−06	7.01E−03	−3.13E−05	9.72E−05	692	−4.14E−04	7.04E−03	−1.21E−04	1.00E−04
418	1.95E−05	7.85E−03	−3.17E−05	1.18E−04	556	−1.09E−05	7.04E−03	−2.75E−05	9.80E−05	694	−4.00E−04	7.03E−03	−1.39E−04	1.00E−04
420	1.31E−05	7.95E−03	−3.19E−05	1.19E−04	558	−1.87E−05	7.05E−03	−2.49E−05	9.84E−05	696	−3.52E−04	7.15E−03	−1.57E−04	9.78E−05
422	8.85E−06	8.02E−03	−3.21E−05	1.21E−04	560	−2.68E−05	7.00E−03	−2.63E−05	9.86E−05	698	−2.19E−04	7.19E−03	−1.69E−04	1.02E−04
424	5.62E−06	8.12E−03	−3.23E−05	1.22E−04	562	−3.46E−05	7.05E−03	−2.85E−05	1.02E−04	700	−2.83E−05	7.13E−03	−1.91E−04	1.00E−04
426	2.30E−06	8.16E−03	−3.24E−05	1.21E−04	564	−4.22E−05	7.09E−03	−2.90E−05	9.73E−05	702	1.99E−04	7.08E−03	−2.10E−04	9.80E−05
428	3.14E−07	8.22E−03	−3.26E−05	1.22E−04	566	−4.86E−05	7.09E−03	−2.96E−05	9.76E−05	704	4.58E−04	6.97E−03	−2.24E−04	1.01E−04
430	1.75E−06	8.19E−03	−3.28E−05	1.21E−04	568	−5.31E−05	7.04E−03	−3.18E−05	9.87E−05	706	7.94E−04	6.93E−03	−2.36E−04	9.53E−05
432	5.75E−06	8.14E−03	−3.30E−05	1.20E−04	570	−5.59E−05	7.01E−03	−3.45E−05	9.89E−05	708	1.22E−03	6.99E−03	−2.60E−04	1.04E−04
434	9.88E−06	8.04E−03	−3.31E−05	1.18E−04	572	−5.59E−05	7.03E−03	−3.65E−05	9.80E−05	710	1.73E−03	6.90E−03	−2.61E−04	1.05E−04
436	1.28E−05	7.92E−03	−3.33E−05	1.18E−04	574	−5.02E−05	7.03E−03	−3.75E−05	9.73E−05	712	2.34E−03	6.94E−03	−2.87E−04	1.08E−04
438	1.40E−05	7.82E−03	−3.34E−05	1.15E−04	576	−3.77E−05	6.97E−03	−4.01E−05	9.88E−05	714	3.01E−03	6.92E−03	−2.93E−04	1.08E−04
440	1.49E−05	7.75E−03	−3.36E−05	1.13E−04	578	−1.68E−05	6.94E−03	−4.33E−05	9.92E−05	716	3.70E−03	6.77E−03	−2.99E−04	1.09E−04
442	1.57E−05	7.68E−03	−3.38E−05	1.14E−04	580	1.43E−05	6.96E−03	−4.50E−05	9.84E−05	718	4.39E−03	6.92E−03	−3.16E−04	1.02E−04
444	1.70E−05	7.63E−03	−3.39E−05	1.12E−04	582	5.65E−05	6.96E−03	−4.59E−05	9.74E−05	720	5.10E−03	6.93E−03	−3.22E−04	1.13E−04
446	1.92E−05	7.54E−03	−3.40E−05	1.12E−04	584	1.06E−04	6.92E−03	−4.92E−05	9.71E−05	722	5.84E−03	7.01E−03	−3.37E−04	1.04E−04
448	2.29E−05	7.46E−03	−3.49E−05	1.12E−04	586	1.58E−04	6.92E−03	−5.27E−05	9.83E−05	724	6.72E−03	6.98E−03	−3.63E−04	1.22E−04
450	2.68E−05	7.38E−03	−3.55E−05	1.12E−04	588	2.11E−04	6.94E−03	−5.22E−05	9.75E−05	726	7.88E−03	6.86E−03	−3.81E−04	1.14E−04
452	2.83E−05	7.30E−03	−3.56E−05	1.12E−04	590	2.68E−04	6.96E−03	−5.11E−05	9.81E−05	728	9.37E−03	6.58E−03	−4.23E−04	1.10E−04
454	2.57E−05	7.20E−03	−3.56E−05	1.08E−04	592	3.37E−04	6.98E−03	−5.45E−05	9.74E−05	730	1.11E−02	6.64E−03	−4.60E−04	1.23E−04
456	2.15E−05	7.10E−03	−3.63E−05	1.08E−04	594	4.23E−04	6.96E−03	−5.87E−05	9.82E−05	732	1.28E−02	6.42E−03	−4.63E−04	1.22E−04
458	1.86E−05	7.04E−03	−3.68E−05	1.06E−04	596	5.29E−04	6.96E−03	−6.05E−05	9.91E−05	734	1.41E−02	6.27E−03	−4.63E−04	1.25E−04
460	1.79E−05	7.00E−03	−3.64E−05	1.06E−04	598	6.54E−04	7.00E−03	−6.12E−05	9.82E−05	736	1.47E−02	6.59E−03	−3.83E−04	1.39E−04
462	1.73E−05	6.96E−03	−3.61E−05	1.05E−04	600	7.85E−04	7.01E−03	−5.75E−05	9.80E−05	738	1.47E−02	6.65E−03	−2.81E−04	1.34E−04
464	1.47E−05	6.98E−03	−3.51E−05	1.05E−04	602	9.02E−04	7.00E−03	−5.15E−05	9.89E−05	740	1.42E−02	6.43E−03	−1.38E−04	1.40E−04
466	1.10E−05	7.01E−03	−3.42E−05	1.04E−04	604	9.86E−04	6.98E−03	−4.21E−05	9.84E−05	742	1.33E−02	6.64E−03	1.10E−05	1.55E−04
468	7.32E−06	7.06E−03	−3.39E−05	1.04E−04	606	1.02E−03	7.03E−03	−2.92E−05	9.82E−05	744	1.22E−02	6.64E−03	1.76E−04	1.49E−04
470	5.42E−06	7.08E−03	−3.50E−05	1.06E−04	608	1.00E−03	7.09E−03	−1.34E−05	9.82E−05	746	1.10E−02	6.55E−03	2.85E−04	1.24E−04
472	4.59E−06	7.11E−03	−3.60E−05	1.05E−04	610	9.45E−04	7.09E−03	−9.97E−07	9.73E−05	748	9.85E−03	6.20E−03	3.69E−04	1.83E−04
474	5.01E−06	7.12E−03	−3.58E−05	1.05E−04	612	8.60E−04	7.04E−03	8.09E−06	9.71E−05	750	8.69E−03	6.43E−03	4.46E−04	1.43E−04
476	6.08E−06	7.16E−03	−3.55E−05	1.05E−04	614	7.74E−04	7.00E−03	1.20E−05	9.82E−05	752	7.57E−03	6.54E−03	5.09E−04	1.60E−04
478	6.93E−06	7.15E−03	−3.48E−05	1.05E−04	616	6.95E−04	7.03E−03	1.54E−05	9.69E−05	754	6.46E−03	6.35E−03	5.08E−04	1.44E−04
480	6.94E−06	7.13E−03	−3.58E−05	1.06E−04	618	6.22E−04	7.09E−03	1.87E−05	9.66E−05	756	5.37E−03	7.33E−03	5.47E−04	1.53E−04
482	5.17E−06	7.08E−03	−3.79E−05	1.03E−04	620	5.44E−04	7.09E−03	1.98E−05	9.59E−05	758	4.33E−03	6.10E−03	5.29E−04	1.69E−04
484	2.88E−06	7.08E−03	−3.99E−05	1.06E−04	622	4.59E−04	7.00E−03	1.73E−05	9.71E−05	760	3.35E−03	6.41E−03	5.35E−04	1.51E−04
486	1.01E−06	7.01E−03	−3.87E−05	1.06E−04	624	3.67E−04	6.96E−03	1.56E−05	9.55E−05	762	2.39E−03	6.68E−03	5.13E−04	1.74E−04
